# The sexual and reproductive healthcare challenges when dealing with female migrants and refugees in low and middle-income countries (a qualitative evidence synthesis)

**DOI:** 10.1186/s12889-024-17916-0

**Published:** 2024-02-19

**Authors:** Tadele Dana Darebo, Mark Spigt, Berhanetsehay Teklewold, Abebe Sorsa Badacho, Niklas Mayer, Meba Teklewold

**Affiliations:** 1 School of Public Health, Wolaita Sodo University, Wolaita Sodo, Ethiopia; 2 Research Institute CAPHRI, Department of Family Medicine, Maastricht University, Maastricht, Netherlands; 3 Maastricht University, Maastricht, Netherlands; 4 General Practice Research Unit, Department of Community Medicine, UiT the Arctic University of Norway, Tromso, Norway; 5 Saint Paul’s Hospital Millenium Medical College, Addis Ababa, Ethiopia; 6 UNHCR Ethiopia, Addis Ababa, Ethiopia

**Keywords:** Migrants, Refugees, Sexual and reproductive health, Person centered care, Low and-middle income countries, Evidence synthesis

## Abstract

**Background:**

Migrants and refugees face unprecedented inequalities in accessing sexual and reproductive health (SRH) in developed and developing countries. Most attention has focused on the rich world perspective, while there are huge numbers of migrants and refugees moving towards less developed countries. This article synthesizes the barriers to proper SRH care from low and middle-income countries perspective.

**Methods:**

We performed a systematic review of articles containing primary source qualitative and quantitative studies with thick qualitative descriptions. Articles from various databases, including PubMed, Science Direct, HINARI, and Google Scholar, published between 2012 and 2022 were included. Because the context differed, we excluded articles dealing with migrants and refugees from low- and middle-income countries living in high-income countries. To select articles, a preferred reporting item for systematic reviews and meta-analyses (PRISMA) was used. The articles’ quality was assessed using the standard QASP checklist. We used a socio-ecological model to investigate barriers at various levels, and thematic analysis was used to identify the strongest themes at each level of the model. This synthesis is registered under PROSPERO number CRD42022341460.

**Results:**

We selected fifteen articles from a total of 985 for the final analysis. The results show that despite the diversity of the participants’ homes and countries of origin, their experiences using SRH services were quite similar. Most female migrants and refugees claimed to have encountered discrimination from service providers, and linguistic and cultural obstacles played a significant role in their experiences. In nations lacking universal healthcare coverage, the cost of care was a barrier to the use of SRH services. Other main obstacles to using SRH services were a lack of knowledge about these programs, worries about privacy, inadequate communication, stigma in the community, and gender-related power imbalances.

**Conclusion:**

To enhance the use of SRH by female migrants and refugees, it is vital to provide person-centered care and involve husbands, parents, in-laws, and communities in SRH coproduction. Training on cultural competency, compassion, and respect must be provided to healthcare personnel. Increasing financial access for migrant and refugee healthcare is crucial, as is meeting their basic requirements.

**Supplementary Information:**

The online version contains supplementary material available at 10.1186/s12889-024-17916-0.

## Introduction

Women and girls accounted for 48% (134.9 million) of all international migrants worldwide in 2020, making up half of all migrants and refugees and in vulnerable humanitarian settings [1]. They usually cannot find a job, do not receive any education, and cannot consult a physician for sexual and reproductive health (SRH) issues [2, 3]. According to the World Health Organization (WHO), migrant and refugee women are at a higher risk of rape, unwanted pregnancy, and unsafe abortion [4]. Because of these circumstances, they also suffer from depression and social isolation more often [5–9]. One of the fundamental rights enshrined in Agenda 2030 for Sustainable Development is access to universal health coverage (UHC), including SRH services to everyone including migrants and refugees [10]. Sexual and reproductive health and rights are one of inalienable human rights according to the international conventions [11], and services encompass the provision of information and assistance in preventing, diagnosing, counseling, treating, and caring for individuals in all matters related to SRH [12]. It is a prerequisite for achieving gender equality as well as good health and well-being. Hence, there is an increasing global need to address the sexual and reproductive health of migrants and refugees [3, 13, 14].

According to evidence from systematic reviews conducted in the developed world, being a migrant or refugee often results in power imbalances to make SRH decisions [15, 16]. Refugees and migrants traveling to developed countries face sociocultural barriers to obtaining and using SRH services [17–21]. These impediments are combined with legal and other traditional power dynamics. Furthermore, they face language barriers, which result in inaccurate information about SRH issues, communication barriers, and feelings of embarrassment when discussing SRH issues [22–24].

Nearly 45 million women were displaced by the end of 2021, and 83% of them hosted in low- and middle-income countries experiencing a humanitarian crisis with urgent SRH needs [25]. Understanding the barriers and needs is critical for improving migrants’ SRH and the development of new interventions [13, 26, 27]. Independent literature identified barriers in Africa [28, 29], Asia [30–32], and Latin America [33, 34] countries. However, there is no systematic qualitative evidence synthesis on SRH utilization barriers for migrants and refugees. This systematic analysis of the existing studies provides a holistic picture of the barriers that would inform targeted interventions at international or local levels. The findings will help program managers, policymakers, and healthcare providers develop appropriate tools to address barriers to SRH utilization in low and middle-income countries.

## Methods

**Fig. 1:**
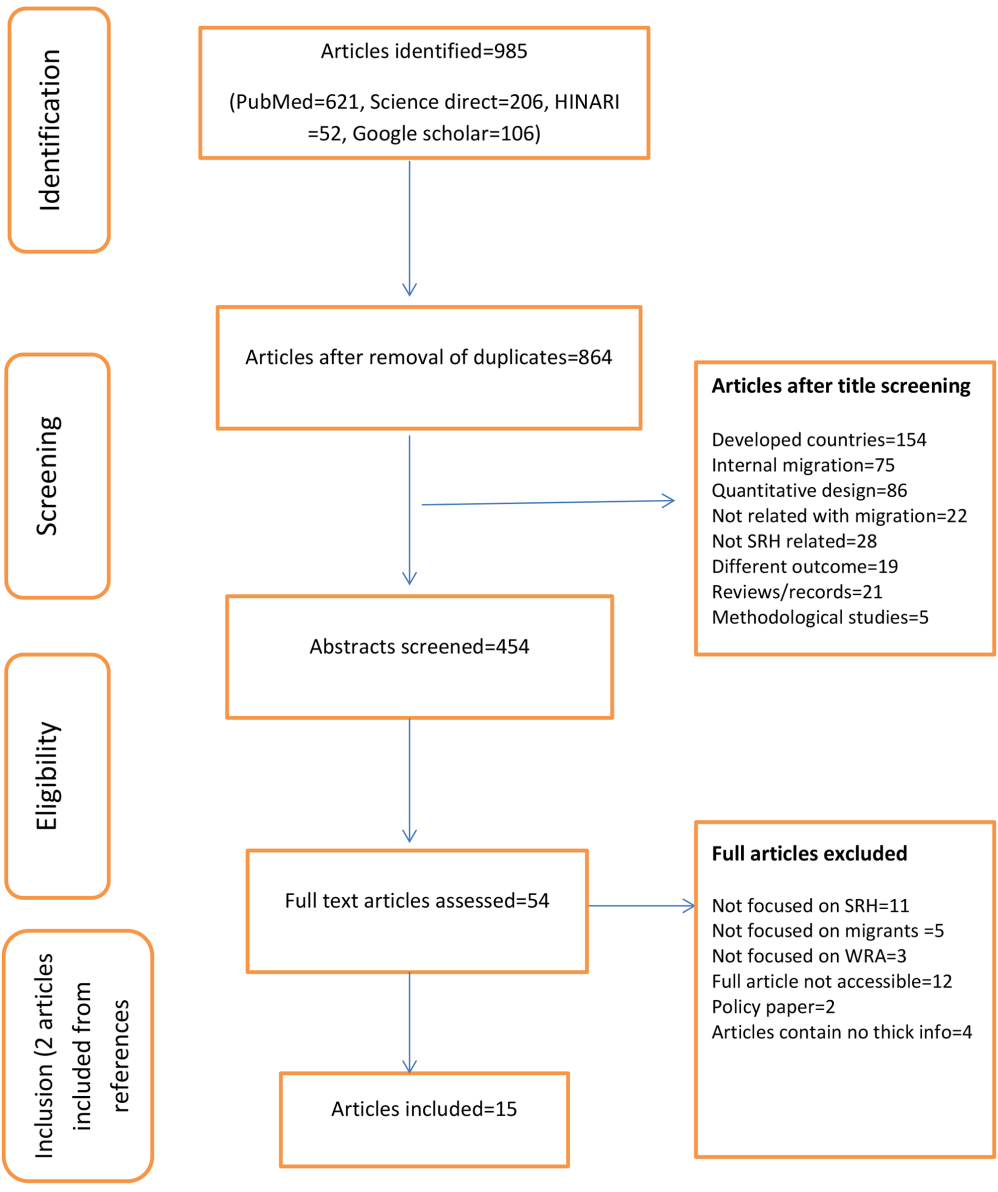
Flow diagram to select article for the systematic synthesis to identify the sexual and reproductive health challenges of female migrants in low income settings

A preferred reporting item for systematic reviews and meta-analyses (PRISMA) was used to select the articles [35] (Fig. 1). All qualitative and quantitative studies with sufficient qualitative descriptions published between 2011 and 2022 were included from various databases such as PubMed, Science Direct, HINARI, and Google Scholar. We applied both textword and MeSH terms while searching, to increase the chance of getting the potential articles. The four main categories of the search were related to (1) barriers/facilitators, (2) SRH services, (3) migrants/refugees, and (4) women/girls. For example, we used the textword to search for barriers as (e.g. “barriers” OR “facilitators” OR “problems” OR ”qualitative research” OR “exploration”) AND sexual and reproductive health service as (“reproductive health” OR “reproductive health service*” OR “reproductive health utilization” OR “sexual health service*” OR “contraceptives*” OR “antenatal utilization” OR “obstetric care utilization”) AND the study population were searched as (e.g. “migrant*” OR “refugee*“ OR “transients” OR “refugee camps” AND females were searched as (e.g. “women” OR “female” OR “young women” OR “pregnant women”) (Appendix 1). We searched broadly to capture recognized or unrecognized, resettled, registered, or unregistered migrants and refugees.

### Inclusion and exclusion criteria

In this study, only peer-reviewed articles describing studies in low and middle-income countries (LMIC) that were published in the last ten years from 2012 up to 2022 were included. The main phenomenon we were interested in was migrant women’s and girls’ experiences related to sexual and reproductive services utilization. The publication language had to be English. Those articles that contained migrants described as refugees, unregistered migrants, and asylum seekers, were part of this study. The perspectives of care providers were also included in this study. Qualitative and quantitative studies with thick descriptions of the qualitative findings were included.

We excluded articles that dealt with migrants or refugees from low and middle-income countries moving to or residing in high-income countries since the context is expected to be different. We also excluded purely quantitative studies, letters, case reports, reviews, commentaries, books, protocols, theses, and editorials. Internal migration was also beyond the scope of this study. Relevant references were also searched to include potential studies in this synthesis.

### Study selection and data extraction

Titles and abstracts were screened based on the inclusion and exclusion criteria by two researchers independently (TDD and ASB). Then, double-screening of the full text of potentially relevant sources was done. Finally, team members discussed any disagreements concerning eligibility. All qualitative data related to women’s experiences of reproductive health and healthcare utilization barriers were extracted using a standardized form. The quality of the articles was checked by using the Critical Appraisal Skills Programme (QASP) by Oxford University [36]. The Mendeley reference manager software was used to record all articles, and duplicates were removed. We did a full-text review of the texts that passed the initial screening. The ENTREQ statement [37], Enhancing Transparency in Reporting the Synthesis of Qualitative Research, was used to extract data. This is a 21-item tool to capture characteristics of the study that were included in the analysis. It contains information such as synthesis methodology, approach to searching, and other parameters required. All articles included in the study provided the relevant information. (Appendix 2). This synthesis is registered under PROSPERO number CRD42022341460.

### Data analysis process

The findings were analyzed using thematic analysis. The data were first placed in an Excel sheet to review the contents of the study, which included study population characteristics, region, publication year, and other contexts such as socio-demographic and cultural aspects of the study area. We developed initial codes, then sub-themes, and themes under each level of the socio-ecological model. We chose to use this model because it demonstrates the complex interplay between individual (e.g., behaviors), social and community (e.g., norms), institutional and systemic (e.g., health services, education), and structural (e.g., laws, protection mechanisms) factors that affect the health and wellbeing of migrants and refugees [38, 39]. For some sub-themes that needed further elaboration, quotations were also extracted from the primary studies. Open code 4.02 was used for data management.

## Results

We applied the socio-ecological framework that includes four levels of analysis: individual, social and community, institutional and health system, and structural levels [40, 41]. A qualitative content analysis was used to extract data and organize sub-themes. Barriers were analyzed to identify the strongest themes at each level. From the selected studies, 12 mentioned strong individual level barriers [28–30, 34, 42–49], 11 stated barriers at the social and community level [28–31, 34, 42, 45, 46, 48, 50, 51], 10 mentioned issues related to the institutional and health care level [28, 30, 31, 34, 43, 46, 48–51], and 9 mentioned structural level barriers [28, 30, 31, 45–48, 50, 51]. In eight studies, we identified all of the four-level barriers to SRH utilization among migrants and refugees.

### Individual level barriers

The strongest themes in the individual level barriers are communication, and SRH knowledge and perception related ones. Subthemes within the former category encompass patient-provider communication, communication between spouses, and parent-adolescent communication, while the latter category includes subthemes such as awareness about the availability and use of services, misinformation, low-risk perception of vulnerability, poor self-perception, reluctance to use services due to shame, and fear of side effects.

Communication is the strongest aspect of individual-level barriers. It is an important aspect of establishing a connection between service providers and clients, and in the absence of effective communication, service provision becomes almost impossible [15]. It is through communication that health complaints, symptoms, diagnosis, treatment, follow-up, and prognosis are established. Refugees and migrants face more challenges because they come to a new host country that may speak a different language [15, 52]. Communication is not only difficult for them, but it is also one of the most problematic tasks for medical personnel. Both the client and provider present it as one of the main barriers to utilizing SRH services [44, 51]. Parent-daughter and couples communication is another barrier related to SRH utilization among refugees and migrants in low and middle-income countries [30, 34, 44, 46].

A study on reproductive health among Venezuelan migrant women in Brazil identified communication as the main barrier to maternal antenatal and childbirth services. They expressed that not being fluent in Portuguese resulted in discrimination in the healthcare system and placed them in a position where they were unable to get enough attention from the healthcare providers. Because of the communication gap, they faced further challenges of long waits, contributing to service dissatisfaction and posing future barriers to seeking the service [34].

Parent-adolescent communication is another barrier to SRH utilization, especially among youth. In many Arab countries, communication about SRH remains taboo. Most parents reserve such information until marriage and it is usually incomplete [7, 30, 53]. A study on healthcare provider and educator perspectives among Syrian refugees on adolescent SRH in Lebanon [23] identified inadequate SRH-related communication between parents and daughters. They usually block such communications with stress, shame, discomfort, and stigma which results in barriers to dialogue and utilization.

“Parents don’t bring up these topics (with their children). They’ll tell you: ‘You’re opening up my daughter’s eyes to something bad!’ Or they’ll say: ‘She won’t be thinking about these things until she hears about them.’ But who says she’s not thinking about this? Maybe she is thinking about these topics but she is too afraid (to discuss with her parents)? If a mother cannot educate her daughter (on this issue) then she should ensure that her daughter is receiving the correct information elsewhere.” (High-school teacher, IDI).

A similar study in Bihar, India [44] identified poor spousal communication as the barrier to not using family planning among migrants. The study identified poor couple communication regarding contraceptive use, and they placed the topic in the backseat, while other issues like household needs, children, and family issues dominate the discussions. Migrants perceive that contraceptive use is usually on the table, and husbands have little or no interest in bringing it forth. In such cases, women remain either silent or in fear of provoking any marital conflict by bringing issues to the front.

“We do not bring up FP issues when husbands are at home, this might bring unnecessary conflict. We only do what they ask us to do.” (Woman, age 20).

Other individual-level barriers include sexual and reproductive health knowledge-related factors such as lack of awareness about the availability and use of the service [28, 30, 44, 48], misinformation [28, 34], low-risk perception of vulnerability [30, 45, 46], poor self-perception [28, 45], shame to use the service and fear of side effects [30, 44]. A study on the use of family planning services by Syrian women in a refugee camp in Jordan by West et al. [48], identified misinformation and poor SRH knowledge of refugees hindered the use of the services. Although family planning is good for their health and the well-being of their children, most mothers tend to not use the service because of negative thoughts and misinformation. Participants expressed their concern that byh using modern contraceptives they may lose their fertility. As expressed by one participant in that study,

“We believe that after the first child it’s preferable… not to have (unspecified) contraception methods because we think… maybe we won’t be able to have more children… Some women have been sterile after they used contraception.” (Participant 7, FGD).

“They said it (the OCP) might cause me not to have children anymore.” (Participant 6, FGD).

Another woman expressed a lack of knowledge of where to get the service. She stated,

“The most important (problem) is that people don’t know about the contraceptive methods and where to get them… I don’t know if there are any kinds of (FP) services here and where… no one told me…. nobody cares.” (Participant 9, FGD).

Another study among migrants on the Guatemala-Mexico border identified poor information about health systems regarding SRH. Factors such as contraceptive misinformation, lack of information on access to barrier and non-barrier contraceptives, presence of cervical screening services, and lack of sex education played a role as barriers to SRH utilization [47]. On the other hand, knowledge of the availability and accessibility of the service played a key role in utilizing the service. On the contrary, refugees in Uganda mentioned a lack of awareness about the availability of services, low self-perception, fear, shame, and anxiety as barriers to service utilization [28]. Participants stated in that study,

“I have never gone for contraceptives at the health facility” (16-year-old, IDI).

Furthermore, disability, poor life skills, and school dropout are barriers to SRH utilization, particularly among young immigrants and refugees [30, 41, 42, 54].

### Social and community-level barriers

The second category of barriers in the socio-ecological model deals with social and community-related factors. Gender-based violence and decision-making are the main themes under this category. Under the first theme, discrimination towards women in seeking care and gender-based violence; and under decision making theme lack of male involvement in seeking SRH care and gender-related traditional power dynamics play an enormous role. This has been observed among young girls in Uganda, Syrian migrants in Lebanon, Venezuelan migrants in Brazil, and migrant women in Malaysia, Cambodia, Laos, Thailand, and Vietnam [28, 30, 31, 34, 49]. This shows that regardless of geographical variation and cultural differences, gender-based violence and power imbalance still play a key role as a barrier to using SRH services among migrants, immigrants and refugees in LMICs. A study by Fahme et al. among Syrian refugee girls in Lebanon [30], found that men have an overwhelming power to influence women on whether to take family planning methods or end it based on their (mis)conceptions. Women often have no choice but to comply with the male’s opinion, without question. A healthcare provider stated,

“We have had women coming (to the clinic) several days after getting Implanon requesting that it be removed because their husbands have heard that it causes cancer, or that it can migrate under the skin and embolize to the heart. Even if their husbands were initially accepting of the contraception, they may have heard from their friends or others that it poses health risks to the woman.” (Midwife, FGD).

Sexual and reproductive service utilization decision-making is another challenge. This includes denial of services, negative attitude to abortion care, husbands’ sole decision, and lack of self-right which all play a key role as a barrier to SRH service utilization. When only husbands decide to use a male structure of power, it results in bad maternal and child health outcomes, including morbidity and mortality [30, 45]. In places where a woman’s decision-making is severely limited, adverse health outcomes are inevitable. The decision on the number of children and timing of pregnancy is often determined by the husbands. A healthcare provider noticed,

“They (Rohingya) want more children, their husbands want more children. He wouldn’t allow these things (family planning). And their religious mindset. And they are totally illiterate, they do not know about family planning.” (Paramedic, KII).

A young migrant in the refugee camp of Uganda [28], also stated,

“I have never gone for contraceptives at the health facility. I only use the natural method my husband has told me. But I have plans of using one of the family planning methods.” (16 years old, IDI).

### Institutional and health system-level barriers

Under the institutional and health system level, service quality (lack of effective access and high cost of the service) and professional competency (compassion and poor policy knowledge) are the main barriers to utilize SRH services. The main problems under the service quality are lack of effective access related to the high costs of the services, the absence of 24-hour/7 day services, distance to the facility, lack of timely service, lack of health care resources, lack of health insurance, unavailability of suitable spaces to learn about SRH, limited options of services, poor satisfaction, and sustainability-related problems [44–46]. Refugees and migrants who find themselves in impoverished conditions ca. not afford to pay for services, including medical costs Different studies in Lebanon, Jordan, Malaysia, and Thailand have indicated that the costs of services was the main challenge to accessing maternal or other reproductive health services [9, 23, 37, 38, 40, 41].

A study on undocumented Myanmar migrants in Thailand found that migrants who need emergency services, face unprecedented challenges related to the cost of the service to get a cesarean section delivery [51]. One of the healthcare providers working in the hospital stated:

“In case of critical patients transferred to us who need to have emergency operations to give birth, the cost will be high. Even if we have a few cases, the expenses of the obstetric and newborn sections will be the highest amount when compared with other sections of the hospital” (HCP, IDI).

Another study on Syrian migrant girls in Lebanon [30] identified the high amount of medical costs as a barrier to accessing and using the service.

“I went to a hospital here, but no one helped us. I spent three days in the hospital in Saida (Lebanon), and no one helped us. The medical expenses were very high, and you are aware of our situation here. I went back to Syria to be treated.” (young girl, IDI).

Besides medical costs, poor accessibility and service quality play a major role. Poor quality of the service including lack of needed resources and the absence of 24/7 services was the main challenge among migrants and refugees in Uganda [28], Bangladesh [45], Jordan [48], Cambodia, Laos, Thailand, and Vietnam [49]. In places where service quality and accessibility are not ensured, they face SRH problems that put their lives and future at risk, besides their general frantic life conditions [8, 9, 31, 42, 50].

Healthcare professionals are the persons who should provide appropriate and effective healthcare. However, a lack of compassion can deter access and the use of the services. With refugees, their role is more important since options for different health care and professionals are very limited. Lack of female healthcare workers, denial of service based on marital status, discrimination, inhumane treatment, lack of confidence to provide the service, language barrier, and poor skills and policy knowledge were common barriers. Healthcare in turn often complains about burnout and work overload [28, 30, 43, 44, 48, 50, 51].

A study on the needs and priorities of Syrian refugees in Jordan by Al-Rousan et al. identified highly disrespectful and humiliating treatment from the healthcare providers, which is against human rights [50], and on the Mexico-Guatemala border, discriminatory treatment of foreign immigrants was observed [47]. In such places, migrants usually become liable to pay a high cost in search of better attention and treatment from private clinics, which puts them under greater financial strain [47, 50].

In most studies, refugees and migrants often complain about the absence of female healthcare professionals, which blocks service utilization. Women and girls, particularly in Middle Eastern countries where Syrians have sought refuge, are embarrassed to be treated by male doctors [30, 50]. The behavioral dimension of health care professionals and the gender of the care provider plays a key role in service utilization. Refugees and immigrants question the skills and confidence of healthcare providers in emergency settings. They compare and contrast with the physicians they used to get treatment in their home countries where they received much attention and visited preferred medical personnel [21, 49, 51, 55].

Sometimes health care professionals ignore the needs of unmarried women and girls who need SRH services. One of the migrant women on the Thailand-Myanmar border [46] complained about the denial of service because of her marital status:

“They seem to only give medications and condoms to married couples. If some of them are still in school, they wouldn’t be given anything to prevent pregnancy.” (pregnant refugee, IDI).

Healthcare practitioners in many studies complain about work overload and burnout because of the high number of clients in their settings. They believe this will also play a key role as a barrier to providing quality SRH services to refugees [45, 50].

### Structural level barriers

Structural barriers are those that operate at the macro level in the socio-ecological model. Legal and policy-related barriers are the main challenge blocking migrants not to access services, lack of health insurance, mandatory seeking of documents to provide services, and policy knowledge of gaps by health care providers. These factors work alone or in combination in many studies in this synthesis. In some countries, SRH services are not accessible to migrants merely because of their migration status, whereas others seek documentation completion before rendering essential care that is needed for their survival. For example, women were denied comprehensive abortion care because of their legal status in humanitarian settings in Bangladesh [45] and stripped of their right to get SRH services in Malaysia because of employment contract clauses [56]. The challenges include also discriminatory health Policies, discriminatory prohibition of pregnancy for migrant women, compulsory health screening, denial of marriage for low-skilled professionals, and denying of family planning services under the pretext of preventing promiscuity [31, 56, 57]. Such barriers forced women to seek care from illegal and unsafe sources putting their health in a problematic position [9]. A medical doctor expressed the condition of migrant pregnant women as;

“They will automatically be illegal migrants because the moment they are pregnant, they will lose their visa and if they lose their visa, they become illegal migrants. But somehow, many of them do deliver locally.” (private GP, KII).

In Lebanon, husbands were prohibited from accompanying their pregnant wives because of the clinic policies deterring them from attending the service provision [30]. Such policies discourage not only women from attending future service but also create mistrust and negative attitude towards care and medical personnel. The study in Malaysia has identified the worst case of reporting to the legal authorities for custody when women seek emergency lifesaving services [31].

Other structural factors include traditional and cultural barriers hindering SRH service utilization. These include cultural and social norms, myths, and stereotyping of young girls [28, 34, 46, 51]. Although universal human needs transcend cultures, cultural barriers still pose a significant burden among refugees in Ethiopia [29], Kenya, Uganda, Nepal [42], Malaysia [31], Lebanon [30], and Jordan [50].

## Discussion

This synthesis provides evidence about socio-ecological determinants that preclude women in humanitarian settings from accessing and utilizing services to realize their sexual and reproductive health and their right to the enjoyment of the highest standard of health. According to our findings, migration and refugee status are existing problems in many developing countries, and despite geographic and cultural differences, they face similar barriers to service utilization from the individual to the policy level, which primarily include communication-related barriers, gender-based violence, and decision-making, care quality and compassion and legal barriers in the host countries. Other main causes of poor SRH among refugees in various countries were confirmed by the findings [12, 17, 58, 59]. Our synthesis supplements additional knowledge on how cross-cutting barriers such as basic needs and person centerdness affect the utilization of SRH services and the well-being of women and girls. Training on SRHR, improving access to care, and compassion and communication are the cross-cutting facilitators of SRH service utilization for female refugees in developing countries. Hence, the discussions will focus on those facilitators of SRH service utilization.

### Availing person centered care (PCC) for migrants and refugees in LMICs

Every women including those in the humanitarian context have the right to get a quality SRH care [10, 60]. The quality of care framework by the WHO for SRH places particular emphasis on the experience of care, which includes aspects such as communication, respect and dignity, and emotional support in their specific cultural context [4, 61, 62]. These person-centered factors often influence patients’ opinions about the value of the care them receive and their satisfaction with services. The effectiveness of health systems in meeting clients expectations and their level of trust are also reflected in the perceptions of the quality of care. These person-centered attributes also have an impact on treatment outcomes and on future demand for services [61].

In the context of person centered healthcare, communication is regarded as a critical starting point for establishing trust between the service provider and the client. Communication and linguistic barriers make it problematic for migrants to steer the healthcare system and restrict healthcare personnel from providing proper services to migrants, which reduces the effectiveness of initiatives of health promotion aimed at them [15, 63]. For example, migrants’ inability to effectively explain illness signs and symptoms may reduce the likelihood of syndromic infection detection, resulting in insufficient HIV and STI treatment [64, 65]. Numerous studies have shown that in order to improve the experience and usage of services for migrants and refugees, health practitioners must be culturally competent and including language proficiency [17, 66, 67]. These skills can reduce communication hurdles caused by linguistic and cultural barriers, engage sensitively with various community values, and address perceived and/or experienced discrimination against migrants and refugees by service providers. We advise that curricula for on-the-job continuous professional development or regular training for health care professionals should include cultural competency. In this context, cultural competency includes the understanding of language, cultural safety, cultural awareness, and cultural sensitivity among health workers in addition to honoring cultural values.

### Ensuring rights to healthcare resources and financial means

Direct financial barriers are created by out-of-pocket payment requirements, particularly for female refugees and migrants in LMICs [5, 31, 41, 68]. Securing Sexual and Reproductive Health (SRH) services for undocumented migrants continues to pose a challenge in achieving Universal Health Coverage (UHC), particularly in many low and middle-income countries. Undocumented women in Thailand, Mynamar, and Turkey encounter additional obstacles in obtaining SRH information, family planning services, antenatal, and emergency obstetric services within those contexts [51, 69, 70].Indirect financial barriers may include transportation and housing costs [47]. Moreover, in both developing and wealthy countries, lack of human resources and financial constraints have been recognized as barriers to improving access to and use of SRH services [60, 71, 72]. Governmental and nongovernmental organizations have often raised concerns about financial issues. Lack of funding for SRH among migrants and refugees leads to delays in necessary diagnosis and treatment [2, 12]. For example, low levels of HIV testing among migrants from LMICs are caused by a lack of funding for migrant health, especially for preventive care [73]. Overcoming administrative barriers to accessing care can be a barrier for both refugees and providers. For example, criteria such as proof of residence raise questions about the eligibility of refugees and service providers [74]. Furthermore, it is a challenge for professionals to decide what services can be provided, as different categories of refugees have different entitlements. Even when legally permitted, administrative and financial barriers may limit access to care [31, 66]. Ensuring financial access and support could increase the uptake of services by migrant and refugee women in low- and middle-income countries. Policy guidelines should also take into account any administrative barriers imposed by the host country. In most countries, this gap exists. Countries need to respect laws in humanitarian settings in order to define a common ground that works for all [10, 60, 75].

### The need for promoting awareness and education among men and boys regarding sexual and reproductive health and gender equity

Key decisions about SRH use are made by husbands and in-laws, leaving women with no choice but to consent to avoid punishment and social stigma [44]. Many policies and regulations in LMICs fail to address the different forms of violence that people may face in their destination countries, as opposed to their countries of origin. Studies show that sexual assault, forced sex, transactional sex and other forms of sexual exploitation are very common. Some research suggests that partners or family members may be the initiators of physical or sexual abuse [74, 76]. Assessing sexual exploitation by family members or close relatives can be challenging as girls may be reluctant to come forward and report such incidents. However, we believe that in addition to sexual exploitation and harassment by their partners, a significant proportion of girls are also victimized by family members and close relatives. This finding has been reported in other non-humanitarian [10, 77]. Therefore, we suggest promoting awareness and education among men and boys regarding sexual and reproductive health and gender equity could contribute signifincatly in mitigating those challenges. In addition to addressing the needs of women and girls gender based violence as part of minimum initial package, an in-depth exploration of the problem is required.

### Availing basic services and inclusive cultural contexts

Addressing the needs of migrants and refugees goes beyond the health aspect, and other stakeholders should be involved in meeting the basic needs. There are minimum initial service package outlined by UNHCR, however in most humanitarian settings, people suffer due to lack of basic services that may obscure their priority to SRH need [60].

Cultural differences between migrants and refugees, and members of host communities influence the ease of access to and use of services. Studies have shown that uptake of SRH services is significantly affected by stigma and prejudice based on gender, migration status and other environmental factors [17, 20]. In addition, numerous studies have shown that migrant and refugee communities often stigmatize young people seeking SRH services [17, 78, 79]. In Sweden, a culturally tailored SRH education programme for refugee family members, including husbands, was provided during the settlement phase, and an evaluation found that it increased their understanding of sexual health and gave them the confidence to use the health system [22]. Low- and middle-income countries should benefit from such initiatives for migrant women and their families, ensuring sensitivity to the diversity of local values and attitudes.

The study among undocumented immigrant women in Turkey revealed that some women do not go to the hospital even during childbirth due to the fear of deportation [70]. Such conditions could lead to maternal and fetal morbidity and mortality. For this reason, it is important to draw lessons from commendable approaches taken by authorities in the United States and Northern European countries, where undocumented immigrant women benefit from preventive reproductive health programs without being reported [75, 80, 81].

Furthermore, to develop initiatives that destigmatize sexual health issues and the use of services by young migrants, health system interventions should focus on community members, religious and faith leaders, and multicultural groups [82, 83]. Mechanisms that engage community members in the co-production of healthy SRH should be put in place to improve the well-being of migrants and refugees in LMICs.

### Strength and weakness

The use of the socio-ecological model provides a better understanding of the barriers across countries, including institutional and structural barriers. This synthesis critically appraised primary articles, and we included participants in refugee camps as well as those outside refugee camps. The synthesis provided priority areas for service packages in the health sector and beyond, recognizing the need for a multi-stakeholder approach in low- and middle-income countries. However, the weakness of this study is that, despite our best efforts to conduct a comprehensive search, some studies were not included, publications were limited to the English language, and the time period of only the last ten years may have missed some relevant qualitative data. The focus of current study is among cis-straight women. We also acknowledge that studies may not capture all issues as many women and girls are reluctant to disclose some of the challenges they face due to shame and fear, which is a common culture in many low and middle-income countries.

## Conclusion and recommendations

Optimizing person-centered care, ensuring access to health resources and financing, educating husbands and communities on gender equality, and providing basic services in an inclusive context are the four areas that need intervention to improve SRH uptake among female migrants and refugees, including unregistered ones in low and middle income countries.

Evidence-based SRH services should be made available to promote person-centered care, provide appropriate language support, respect their dignity, and maintain privacy and confidentiality. In particular, husbands and opinion leaders such as religious leaders and family members should be educated about sexual and reproductive health and rights. Further research is also needed to identify the impact of these structural inequalities, such as rights-based approaches to improving SRH for refugees and migrants. In most situations, research into different norms, power dynamics and political prioritization is also important to understand why SRHR remains a deprioritized issue among refugees and migrants.

Nations should establish and communicate healthcare accessibility measures to attain Universal Health Coverage (UHC), extending the right to health for undocumented individuals. This emphasizes the principle that everyone, irrespective of their migration status, should have the ability to avail themselves of the necessary services.

Non-health sectors need to overcome significant structural barriers to SRH. In addition, SRHR policies for migrants need to be broadened to cover incidents such as sexual assault and challenge the culture of gender-based violence. Responses to SRHR should be based on the recognition that refugees and migrants need adequate health systems and legal protections as they are vulnerable in their countries of origin, while travelling and at their final destination. If the right to health is to be maintained, preserved and fully realized in times of need, curative and preventive SRH services for migrants, especially migrants and refugees, must be adequately resourced.

### Electronic supplementary material

Below is the link to the electronic supplementary material.

Supplementary Material 1

Supplementary Material 2

Supplementary Material 3

Supplementary Material 4

Supplementary Material 5

## Data Availability

All data generated or analysed during this study are included in this published article [and its supplementary information files].

## References

[1] Stock IM. United Nations Department of Economic and Social Affairs, Population Division (2020). 2020.

[2] Brandon Chen YY. International migrants’ right to sexual and reproductive health care. Int J Gynaecol Obstet off Organ Int Fed Gynaecol Obstet. 2022. 10.1002/ijgo.14149 35187657

[3] Duarte-Gómez MB, Cuadra-Hernández SM, Ruiz-Rodríguez M, Arredondo A, Cortés-Gil JD (2018). Challenges of health services related to the population displaced by violence in Mexico. Rev Saude Publica.

[4] WHO. WHO recommendations on adolescent sexual and reproductive health and rights [Internet]. Geneva: World Health Organization.; 2018. Licence: CC BY-NC-SA 3.0 IGO. 2018. 1–88 p. Available from: https://apps.who.int/iris/bitstream/handle/10665/275374/9789241514606-eng.pdf?ua=1.

[5] Egli-Gany D, Aftab W, Hawkes S, Abu-Raddad L, Buse K, Rabbani F et al. The social and structural determinants of sexual and reproductive health and rights in migrants and refugees: a systematic review of reviews. East Mediterr heal J = La Rev Sante La Mediterr Orient = Al-Majallah Al-Sihhiyah Li-Sharq Al-Mutawassit. 2021;27(12):1203–13. 10.26719/emhj.20.101 PMC7616978 35137389

[6] Gurnah K, Khoshnood K, Bradley E, Yuan C (2011). Lost in translation: reproductive health care experiences of Somali Bantu women in Hartford, Connecticut. J Midwifery Womens Health.

[7] Willey SM, Blackmore RP, Gibson-Helm ME, Ali R, Boyd LM, McBride J, et al. “If you don’t ask? you don’t tell”: Refugee women’s perspectives on perinatal mental health screening. Women Birth. 2020 Sep;33(5):e429–37. 10.1016/j.wombi.2019.10.003 31759865

[8] Leeners B, Bieli S, Huang D, Tschudin S (2017). Why prevention of repeat abortion is so challenging: psychosocial characteristics of women at risk. Eur J Contracept Reprod Heal care off J Eur Soc Contracept.

[9] Harris M, Humphries K, Nabb J (2006). Delivering care for women seeking refuge. RCM Midwives.

[10] Greene ME, Patton G, Kieny MP, Evans DB et al. International Federation of Gynecology and Obtetrics (FIGO), United Nations Population Fund (UNFPA),. Sexual and reproductive health and rights: an essential element of universal health coverage. J Adolesc Heal [Internet]. 2019;19(1):S1–2. Available from: 10.1080/17441692.2014.986169%0A

[11] United Nations. Convention on the Elimination of All Forms of Discrimination against Women [Internet]. Available from: https://www.un.org/womenwatch/daw/cedaw/. 12346612

[12] Egli D, Aftab W, Hawkes S, Abu-Raddad L, Buse K, Rabbani F (2021). The social and structural determinants of sexual and reproductive health and rights in migrants and refugees: a systematic review of reviews. East Mediterr Heal J.

[13] Wickramage K, Vearey J, Zwi AB, Robinson C, Knipper M. Migration and health: a global public health research priority. BMC Public Health. 2018;18(1). 10.1186/s12889-018-5932-5 PMC6083569 30089475

[14] Castañeda H, Holmes SM, Madrigal DS, Young MEDT, Beyeler N, Quesada J. Immigration as a social determinant of health. Annual Review of Public Health. Volume 36. Annual Reviews Inc.; 2015. pp. 375–92. 10.1146/annurev-publhealth-032013-182419 25494053

[15] Brandenberger J, Tylleskär T, Sontag K, Peterhans B, Ritz N (2019). A systematic literature review of reported challenges in health care delivery to migrants and refugees in high-income countries-the 3 C model. BMC Public Health.

[16] Ali M, Cordero JP, Khan F, Folz R (2019). Leaving no one behind: a scoping review on the provision of sexual and reproductive health care to nomadic populations. BMC Womens Health.

[17] Maheen H, Chalmers K, Khaw S, McMichael C (2021). Sexual and reproductive health service utilisation of adolescents and young people from migrant and refugee backgrounds in high-income settings: a qualitative evidence synthesis (QES). Sex Health.

[18] Marques P, Nunes M, Antunes Mda, Heleno L, Dias B (2020). Factors associated with cervical cancer screening participation among migrant women in Europe: a scoping review. Int J Equity Health.

[19] Lang AY, Bartlett R, Robinson T, Boyle JA (2020). Perspectives on preconception health among migrant women in Australia: a qualitative study. Women Birth.

[20] Almeida LM, Caldas JP, Ayres-de-Campos D, Dias S (2014). Assessing maternal healthcare inequities among migrants: a qualitative study. Cad Saude Publica.

[21] Rade DA, Crawford G, Lobo R, Gray C, Brown G. Sexual health help-seeking behavior among migrants from Sub-saharan Africa and South East Asia living in High Income countries: a systematic review. Int J Environ Res Public Health. 2018;15(7). 10.3390/ijerph15071311 PMC6069090 29932158

[22] Oscarsson MG, Stevenson-Ågren J (2020). Midwives experiences of caring for immigrant women at antenatal care. Sex Reprod Healthc off J Swedish Assoc Midwives.

[23] Peláez S, Hendricks KN, Merry LA, Gagnon AJ (2017). Challenges newly-arrived migrant women in Montreal face when needing maternity care: Health care professionals’ perspectives. Global Health.

[24] Eslier M, Deneux-Tharaux C, Sauvegrain P, Schmitz T, Luton D, Mandelbrot L (2020). Association between migrant women’s legal status and prenatal care utilization in the PreCARE Cohort. Int J Environ Res Public Health.

[25] UNHRC. Stories behind the numbers. Glob Rep 2021 [Internet]. 2021;Geneva(Swizerland):17–8. Available from: https//reporting.unhcr.org and www.unhcr.org Twitter@UNHCRgov.

[26] (IOM) IO for M. Health of Migrants: Resetting the Agenda - Report of the 2nd Global Consultation. Int Organ Migr [Internet]. 2017;(February):21–3. Available from: https://migrationhealthresearch.iom.int/health-migrants-resetting-agenda-report-2nd-global-consultation-colombo-sri-lanka-21-23-february.

[27] Tomás CC, Oliveira E, Sousa D, Uba-Chupel M, Furtado G, Rocha C et al. Proceedings of the 3rd IPLeiria’s International Health Congress: Leiria, Portugal. 6–7 May 2016. BMC Health Serv Res. 2016;16 Suppl 3(Suppl 3):200. 10.1186/s12913-016-1423-5 PMC4943498 27409075

[28] BuPkuluki A, Kisaakye P, Mwenyango H, Palattiyil G et al. Adolescent sexual behaviour in a refugee setting in UgandBukuluki, P. (2021) ‘Adolescent sexual behaviour in a refugee setting in Uganda.’, Reproductive health, 18(1), p. 131. 10.1186/s12978-021-01181-0.a. Reprod Health. 2021;18(1):131. 10.1186/s12978-021-01181-0 PMC8222959 34167555

[29] Getachew M, Abay M, Zelalem H, Gebremedhin T, Grum T, Bayray A (2018). Magnitude and factors associated with adherence to Iron-folic acid supplementation among pregnant women in Eritrean refugee camps, northern Ethiopia. BMC Pregnancy Childbirth.

[30] Fahme SA, Sieverding M, Abdulrahim S (2021). Sexual and reproductive health of adolescent Syrian refugee girls in Lebanon: a qualitative study of healthcare provider and educator perspectives. Reprod Health.

[31] Loganathan T, Chan ZX, de Smalen AW, Pocock NS. Migrant women’s Access to sexual and Reproductive Health Services in Malaysia: a qualitative study. Int J Environ Res Public Health. 2020;17(15). 10.3390/ijerph17155376 PMC7432037 32722563

[32] Jeffries R, Abdi H, Ali M, Bhuiyan ATMRH, El Shazly M, Harlass S (2021). The health response to the Rohingya refugee crisis post August 2017: reflections from two years of health sector coordination in Cox’s Bazar, Bangladesh. PLoS ONE.

[33] Makuch MY, Osis MJD, Brasil C, de Amorim HSF, Bahamondes L. Reproductive health among Venezuelan migrant women at the north western border of Brazil: A qualitative study. J Migr Heal [Internet]. 2021;4:100060. Available from: https://www.sciencedirect.com/science/article/pii/S2666623521000271. 10.1016/j.jmh.2021.100060 PMC8352082 34405200

[34] Makuch MY, Osis MJD, Brasil C, de Amorim HSF, Bahamondes L (2021). Reproductive health among Venezuelan migrant women at the north western border of Brazil: a qualitative study. J Migr Heal.

[35] Page MJ, McKenzie JE, Bossuyt P, Boutron I, Hoffmann TC, Mulrow CD (2021). The prisma 2020 statement: an updated guideline for reporting systematic reviews. Med Flum.

[36] www.casp-uk.net. 1994;(2018).

[37] Tong A, Flemming K, McInnes E, Oliver S, Craig J (2012). Enhancing transparency in reporting the synthesis of qualitative research: ENTREQ. BMC Med Res Methodol.

[38] Tirado V, Chu J, Hanson C, Ekström AM, Kågesten A (2020). Barriers and facilitators for the sexual and reproductive health and rights of young people in refugee contexts globally: a scoping review. PLoS ONE.

[39] Kaufmann C, Zehetmair C, Jahn R, Marungu R, Cranz A, Kindermann D et al. Maternal mental healthcare needs of refugee women in a State Registration and reception centre in Germany: a descriptive study. Health Soc Care Community. 2021. 10.1111/hsc.13508 34250665

[40] Mengesha ZB, Perz J, Dune T, Ussher J (2017). Refugee and migrant women’s engagement with sexual and reproductive health care in Australia: A socio-ecological analysis of health care professional perspectives. PLoS ONE.

[41] White K, Ocampo M, Scarinci IC (2017). A socio-ecological approach for examining factors related to contraceptive use among recent Latina immigrants in an emerging latino state. Women Health.

[42] Tanabe M, Nagujjah Y, Rimal N, Bukania F, Krause S (2015). Intersecting sexual and reproductive health and disability in humanitarian settings: risks, needs, and capacities of refugees with disabilities in Kenya, Nepal, and Uganda. Sex Disabil.

[43] Davison CM, Watt H, Michael S, Bartels SA (2021). I don’t know if we’ll ever live in harmony: a mixed-methods exploration of the unmet needs of Syrian adolescent girls in protracted displacement in Lebanon. Arch Public Health.

[44] Mukherjee S, Mahapatra B, Saggurti N (2021). Why women do not use contraceptives: exploring the role of male out-migration. PLoS ONE.

[45] Persson M, Larsson EC, Islam NP, Gemzell-Danielsson K, Klingberg-Allvin M (2021). A qualitative study on health care providers’ experiences of providing comprehensive abortion care in Cox’s Bazar, Bangladesh. Confl Health.

[46] Asnong C, Fellmeth G, Plugge E, Wai NS, Pimanpanarak M, Paw MK (2018). Adolescents’ perceptions and experiences of pregnancy in refugee and migrant communities on the Thailand-Myanmar border: a qualitative study. Reprod Health.

[47] Rocha-Jiménez T, Morales-Miranda S, Fernández-Casanueva C, Brouwer KC, Goldenberg SM (2018). Stigma and unmet sexual and reproductive health needs among international migrant sex workers at the Mexico-Guatemala border. Int J Gynaecol Obstet off Organ Int Fed Gynaecol Obstet.

[48] West L, Isotta-Day H, Ba-Break M, Morgan R (2016). Factors in use of family planning services by Syrian women in a refugee camp in Jordan. J Fam Plan Reprod Heal care.

[49] Webber G, Spitzer D, Somrongthong R, Dat TC, Kounnavongsa S (2012). Facilitators and barriers to accessing reproductive health care for migrant beer promoters in Cambodia, Laos, Thailand and Vietnam: a mixed methods study. Global Health.

[50] Al-Rousan T, Schwabkey Z, Jirmanus L, Nelson BD (2018). Health needs and priorities of Syrian refugees in camps and urban settings in Jordan: perspectives of refugees and health care providers. East Mediterr Heal J.

[51] Tschirhart N, Jiraporncharoen W, Thongkhamcharoen R, Yoonut K, Ottersen T, Angkurawaranon C (2021). Including undocumented migrants in universal health coverage: a maternal health case study from the Thailand-Myanmar border. BMC Health Serv Res.

[52] Venters H, Gany F (2011). African immigrant health. J Immigr Minor Heal.

[53] Matin M, LeBaron S (2004). Attitudes toward cervical cancer screening among muslim women: a pilot study. Women Health.

[54] Tanabe M, Greer A, Leigh J, Modi P, Davis WW, Mhote PP (2019). An exploration of gender-based violence in eastern Myanmar in the context of political transition: findings from a qualitative sexual and reproductive health assessment. Sex Reprod Heal Matters.

[55] Kurth E, Jaeger FN, Zemp E, Tschudin S, Bischoff A (2010). Reproductive health care for asylum-seeking women - a challenge for health professionals. BMC Public Health.

[56] Gupta S. Impact of human migration and challenges in reproductive health. Int J Gynecol Obstet. 2015;131.

[57] Steinbrook E, Min MC, Kajeechiwa L, Wiladphaingern J, Paw MK, Pimanpanarak MPJ (2021). Distance matters: barriers to antenatal care and safe childbirth in a migrant population on the Thailand-Myanmar border from 2007 to 2015, a pregnancy cohort study. BMC Pregnancy Childbirth.

[58] Bains S, Sundby J, Lindskog BV, Vangen S, Sørbye IK. Newly arrived migrant women’s experience of Maternity Health Information: a face-to-face Questionnaire Study in Norway. Int J Environ Res Public Health. 2021;18(14). 10.3390/ijerph18147523 PMC8307311 34299974

[59] Kasper A, Mohwinkel L-M, Nowak AC, Kolip P (2022). Maternal health care for refugee women - a qualitative review. Midwifery.

[60] Nabulsi D, Abou Saad M, Ismail H, Doumit MAA, El-Jamil F, Kobeissi L (2021). Minimum initial service package (MISP) for sexual and reproductive health for women in a displacement setting: a narrative review on the Syrian refugee crisis in Lebanon. Reprod Health.

[61] Afulani PA, Diamond-Smith N, Golub G, Sudhinaraset M (2017). Development of a tool to measure person-centered maternity care in developing settings: validation in a rural and urban Kenyan population. Reprod Health.

[62] Fair F, Raben L, Watson H, Vivilaki V, van den Muijsenbergh M, Soltani H (2020). Migrant women’s experiences of pregnancy, childbirth and maternity care in European countries: a systematic review. PLoS ONE.

[63] Schmidt NC, Fargnoli V, Epiney M, Irion O (2018). Barriers to reproductive health care for migrant women in Geneva: a qualitative study. Reprod Health.

[64] Afzal O, Lieber M, Beddoe AM (2020). Reproductive Healthcare Needs of Sex Workers in Rural South Africa: A Community Assessment. Ann Glob Heal.

[65] Falcao J, Ahoua L, Zerbe A, di Mattei P, Baggaley R, Chivurre V (2017). Willingness to use short-term oral pre-exposure prophylaxis (PrEP) by migrant miners and female partners of migrant miners in Mozambique. Cult Health Sex.

[66] Alcaraz Quevedo M, Paredes-Carbonell JJ, Sancho Mestre C, López-Sánchez P, García Moreno JL (2014). Vivas Consuelo D. [Immigrant women care in a health intercultural mediation program]. Rev Esp Salud Publica.

[67] Svensson P, Carlzén K, Agardh A (2017). Exposure to culturally sensitive sexual health information and impact on health literacy: a qualitative study among newly arrived refugee women in Sweden. Cult Health Sex.

[68] Munyaneza Y, Mhlongo EM (2019). Challenges of women refugees in utilising reproductive health services in public health institutions in Durban, KwaZulu-Natal, South Africa. Heal SA = SA Gesondheid.

[69] Tran BX, Vo T, Dang AK, Nguyen QN, Vu GT, Vu LG et al. Characterizing unsafe sexual behavior among factory workers in the Context of Rapid Industrialization in Northern Vietnam. Int J Environ Res Public Health. 2019;16(24). 10.3390/ijerph16245085 PMC6950032 31842473

[70] Mamuk R, Şahin NH (2021). Reproductive health issues of undocumented migrant women living in Istanbul. Eur J Contracept Reprod Heal care off J Eur Soc Contracept.

[71] Eriksen HS, Høy S, Irgens LM, Rasmussen S, Haug K (2020). Social inequalities in the provision of obstetric services in Norway 1967–2009: a population-based cohort study. Eur J Public Health.

[72] Stevenson JE, Oscarsson M (2021). User-centred iterative design to develop an evidence-based communication application for maternity care. Health Inf J.

[73] Padovese V, Farrugia A, Almabrok Ali Ghath S, Rossoni I (2021). Sexually transmitted infections’ epidemiology and knowledge, attitude and practice survey in a set of migrants attending the sexual health clinic in Malta. J Eur Acad Dermatol Venereol.

[74] Johnson JL, Beard J, Evans D (2017). Caring for Refugee Youth in the School setting. NASN Sch Nurse.

[75] IPFF. Sexual rights: an IPPF declaration Who we are. Sex rights an IPPF Declar [Internet]. 2008;1–36. Available from: https://www.ippf.org/sites/default/files/sexualrightsippfdeclaration_1.pdf.

[76] Elnakib S, Hussein SA, Hafez S, Elsallab M, Hunersen K, Metzler J (2021). Drivers and consequences of child marriage in a context of protracted displacement: a qualitative study among Syrian refugees in Egypt. BMC Public Health.

[77] Braatheen S, Rohleder P, Azalde G. Sexual and reproductive health and rights of girls and young women with disabilities. 2017;(July):52.

[78] Korri R, Hess S, Froeschl G, Ivanova O (2021). Sexual and reproductive health of Syrian refugee adolescent girls: a qualitative study using focus group discussions in an urban setting in Lebanon. Reprod Health.

[79] Dias S, Gama A, Rocha C (2010). Perspectives of African and Brazilian immigrant women on sexual and reproductive health. Eur J Contracept Reprod Heal care off J Eur Soc Contracept.

[80] D’agnes T, D’agnes L (1982). Community-based approach to refugee relief: experiences from Thailand. IPPF Med Bull.

[81] Committee opinion no. 627: health care for unauthorized immigrants. Obstet Gynecol. 2015;125(3):755–759. 10.1097/01.AOG.0000461771.63747.37. PMID: 25730255. 10.1097/01.AOG.0000461771.63747.37 25730255

[82] Kolak M, Jensen C, Johansson M (2017). Midwives’ experiences of providing contraception counselling to immigrant women. Sex Reprod Healthc off J Swedish Assoc Midwives.

[83] Gitsels-van der Wal JT, Manniën J, Ghaly MM, Verhoeven PS, Hutton EK, Reinders HS (2014). The role of religion in decision-making on antenatal screening of congenital anomalies: a qualitative study amongst muslim Turkish origin immigrants. Midwifery.

