# Supplementary material for: The sexual and reproductive healthcare challenges when dealing with female migrants and refugees in low and middle-income countries (a qualitative evidence synthesis)
Source: BMC Public Health. 2024 Feb 19;24:520. doi: 10.1186/s12889-024-17916-0 (PMC10877851; doi:10.1186/s12889-024-17916-0)
Supplement: Supplementary file 3 — Supplementary Material 3 [file 12889_2024_17916_MOESM3_ESM.docx]

|  | **Enhancing transparency in reporting the synthesis of qualitative research: the ENTREQ statement** | |  |
| --- | --- | --- | --- |
| **The sexual and reproductive healthcare challenges when dealing with female migrants and refugees in low and middle-income countries (A qualitative evidence synthesis)** | | | |
|  | **No Item Guide and description** |  |  |
| **S.No** | **Item** | **Guide and description** | **Evaluation of review** |
| 1 | Aim | State the research question the synthesis addresses. | To identify barriers to SRH utilization among migrants and refugees in low and middle income countries |
| 2 | Synthesis methodology | Identify the synthesis methodology or theoretical framework which underpins the synthesis, and describe the rationale for choice of methodology (e.g. meta-ethnography, thematic synthesis, critical interpretive synthesis, grounded theory synthesis, realist synthesis, meta-aggregation, meta-study, framework synthesis). | Framework synthesis using socio-ecologicaal model |
| 3 | Approach to searching | Indicate whether the search was pre-planned (comprehensive search strategies to seek all available studies) or iterative (to seek all available concepts until they theoretical saturation is achieved). | pre-planned, comprehensive search strategy applied to seek all available stdies |
| 4 | Inclusion criteria | Specify the inclusion/exclusion criteria (e.g. in terms of population, language, year limits, type of publication, study type). | specified |
| 5 | Data sources | Describe the information sources used (e.g. electronic databases (MEDLINE, EMBASE, CINAHL, psycINFO, Econlit), grey literature databases (digital thesis, policy reports), relevant organisational websites, experts, information specialists, generic web searches (Google Scholar) hand searching, reference lists) and when the searches conducted; provide the rationale for using the data sources | MEDLINE, EMBASE, HINARI, Google scholar, were used |
| 6 | Electronic Search strategy | Describe the literature search (e.g. provide electronic search strategies with population terms, clinical or health topic terms, experiential or social phenomena related terms, filters for qualitative research, and search limits). | specified |
| 7 | Study screening methods | Describe the process of study screening and sifting (e.g. title, abstract and full text review, number of independent reviewers who screened studies) | PRISMA was used |
| 8 | Study characteristics | Present the characteristics of the included studies (e.g. year of publication, country, population, number of participants, data collection, methodology, analysis, research questions). | specified |
| 9 | Study selection results | Identify the number of studies screened and provide reasons for study exclusion (e,g, for comprehensive searching, provide numbers of studies screened and reasons for exclusion indicated in a figure/flowchart; for iterative searching describe reasons for study exclusion and inclusion based on modifications t the research question and/or contribution to theory development). | PRISMA was used |
| 10 | Rationale for appraisal | Describe the rationale and approach used to appraise the included studies or selected findings (e.g. assessment of conduct (validity and robustness), assessment of reporting (transparency), assessment of content and utility of the findings) | Validity was checked using CASP |
| 11 | Appraisal items | State the tools, frameworks and criteria used to appraise the studies or selected findings (e.g. Existing tools: CASP, QARI, COREQ, Mays and Pope [25]; reviewer developed tools; describe the domains assessed: research team, study design, data analysis and interpretations, reporting) | CASP was used |
| 12 | Appraisal process | Indicate whether the appraisal was conducted independently by more than one reviewer and if consensus was required | yes |
| 13 | Appraisal results | Present results of the quality assessment and indicate which articles, if any, were weighted/excluded based on the assessment and give the rationale. | conducted |
| 14 | Data extraction | Indicate which sections of the primary studies were analysed and how were the data extracted from the primary studies? (e.g. all text under the headings “results /conclusions” were extracted electronically and entered into a computer software). | introduction, body, result, conclussion |
| 15 | Software | State the computer software used, if any. | open-code |
| 16 | Number of reviewers | Identify who was involved in coding and analysis. | two reviewers |
| 17 | Coding | Describe the process for coding of data (e.g. line by line coding to search for concepts). | search of concepts |
| 18 | Study comparison | Describe how were comparisons made within and across studies (e.g. subsequent studies were coded into pre-existing concepts, and new concepts were created when deemed necessary) | new concepts were created when deemed necessary |
| 19 | Derivation of themes | Explain whether the process of deriving the themes or constructs was inductive or deductive. | deductive |
| 20 | Quotations | Provide quotations from the primary studies to illustrate themes/constructs, and identify whether the quotations were participant quotations of the author’s interpretation | participants quotations were used |
| 21 | Synthesis output | Present rich, compelling and useful results that go beyond a summary of the primary studies (e.g. new interpretation, models of evidence, conceptual models, analytical framework, development of a new theory or construct). | rich synthesis was done, a new model of evidence |
