# Supplementary material for: The sexual and reproductive healthcare challenges when dealing with female migrants and refugees in low and middle-income countries (a qualitative evidence synthesis)
Source: BMC Public Health. 2024 Feb 19;24:520. doi: 10.1186/s12889-024-17916-0 (PMC10877851; doi:10.1186/s12889-024-17916-0)
Supplement: Supplementary file 4 — Supplementary Material 4 [file 12889_2024_17916_MOESM4_ESM.docx]

**Table: Search strategy to identify articles to study “The sexual and reproductive healthcare challenges when dealing with female migrants and refugees in low and middle-income countries (A qualitative evidence synthesis)”**

| Database used | Search term | Items found |
| --- | --- | --- |
| PubMed | **(((barriers[Text Word]) OR (challenges[Text Word])) OR (facilitators[Text Word])) OR (problems[Text Word])) OR (experiences[Text Word])) OR (hindrances[Text Word])) OR (obstacles[Text Word])) OR (((((“qualitative study”[Text Word]) OR (“qualitative research”[Text Word])) OR (exploration[Text Word])) OR (exploratory study[Text Word])) OR (phenomenological study[Text Word])))) AND ((((((((((((((((((((((((((((reproductive health[Text Word]) OR (reproductive health service*[Text Word])) OR (reproductive health utilization[Text Word])) OR (reproductive care*[Text Word])) OR (reproductive care utilization[Text Word])) OR (reproductive care service*[Text Word])) OR (sexual health[Text Word])) OR (sexual health service*[Text Word])) OR (“sexual health care”[Text Word])) OR (sexual health utilization[Text Word])) OR (sexually transmitted infection[Text Word])) OR (contraceptives[Text Word])) OR (contraceptive utilization[Text Word])) OR (contraceptive care[Text Word])) OR (unmet need[Text Word])) OR (family planning[Text Word])) OR (family planning method[Text Word])) OR (family planning utilization[Text Word])) OR (antenatal care[Text Word])) OR (antenatal utilization[Text Word])) OR (postnatal care[Text Word])) OR (postnatal care utilization[Text Word])) OR (abortion care[Text Word])) OR (abortion service utilization[Text Word])) OR (delivery care[Text Word])) OR (delivery care utilization[Text Word])) OR (delivery service utilization[Text Word])) OR (obstetric care[Text Word]))) AND (((((((((migrant[Text Word]) OR (refugee*[Text Word])) OR (“illegal migrant”[Text Word])) OR (migrants[Text Word] AND transients[Text Word])) OR (transients[Text Word])) OR (foreigner[Text Word])) OR (asylum[Text Word])) OR (immigrant[Text Word])) OR (refugee camps[Text Word]))) AND ((((((((((reproductive age[Text Word]) OR (women[Text Word])) OR (female[Text Word])) OR (adolescent[Text Word])) OR (young women[Text Word])) OR (youth[Text Word])) OR (teenager[Text Word])) OR (young girls[Text Word])) OR (pregnant women[Text Word])) OR (bleeding women[Text Word]))** | 621 |
| **SCIENCE DIRECT** | **Year: 2012-2021**  **Title, abstract, keywords: (Sexual OR reproductive health OR family planning) AND migrants AND (refugees)** | 206 |
| **HINARI** | **(TitleCombined:(sexual OR reproductive OR family planning)) AND (TitleCombined:(Migrants OR refugees)) AND (TitleCombined:(barriers OR facilitators))**  **(The search Include all countries)** | 52 |
| **GOOGLE SCHOLAR** | **allintitle: Barriers facilitators sexual health OR reproductive health OR refugees** | 106 |
