# Supplementary material for: The sexual and reproductive healthcare challenges when dealing with female migrants and refugees in low and middle-income countries (a qualitative evidence synthesis)
Source: BMC Public Health. 2024 Feb 19;24:520. doi: 10.1186/s12889-024-17916-0 (PMC10877851; doi:10.1186/s12889-024-17916-0)
Supplement: Supplementary file 5 — Supplementary Material 5 [file 12889_2024_17916_MOESM5_ESM.docx]

Quality assurance – QASP

|  |  |  | *How to use this appraisal tool: Three broad issues need to be considered when appraising a* | | | | |  |  |  |
| --- | --- | --- | --- | --- | --- | --- | --- | --- | --- | --- |
|  |  |  | *qualitative study:* | *CRITICAL APPRAISAL SKILLS PROGRAM* | | |  |  |  |  |
| SELECTED ARTICLES | 1. Was there a clear statement of the aims of the research? | 2. Is a qualitative methodology appropriate? | 3. Was the research design appropriate to address the aims of the research? | 4. Was the recruitment strategy appropriate to the aims of the research? | 5. Was the data collected in a way that addressed the research issue? | 6. Has the relationship between researcher and participants been adequately considered? | 7. Have ethical issues been taken into consideration? | 8. Was the data analysis sufficiently rigorous? | 9. Is there a clear statement of findings? | 10. How valuable is the research? |
| *Bukuluki A,Kisaakye P,Mwenyango H,Palattiyil G* | YES | YES | YES | YES | YES | YES | YES | YES | YES | VALUABLE |
| *Fahme SA,Sieverding M,Abdulrahim S* | YES | YES | YES | YES | YES | YES | YES | YES | YES | VALUABLE |
| *Makuch MY,Osis MJ,Brasil C,de Amorim HS,Bahamondes L* | YES | YES | YES | YES | YES | YES | YES | YES | YES | VALUABLE |
| *Mukherjee S,Mahapatra B,Saggurti N* | YES | YES | YES | YES | YES | YES | NOT SURE | YES | YES | VALUABLE |
| *Persson M,Larsson EC,Islam NP,Gemzell-Danielsson K,Klingberg-Allvin M* | YES | YES | YES | YES | YES | YES | YES | YES | YES | VALUABLE |
| *Tschirhart N,Jiraporncharoen W,Thongkhamcharoen R,Yoonut K,Ottersen T,Angkurawaranon C* | YES | YES | YES | YES | YES | YES | YES | NOT SURE | YES | VALUABLE |
| *Loganathan T,Chan ZX,de Smalen AW,Pocock NS* | YES | YES | YES | YES | YES | YES | YES | YES | YES | VALUABLE |
| *Asnong C,Fellmeth G,Plugge E,Wai NS,Pimanpanarak M,Paw MK,Charunwatthana P,Nosten F,McGready R* | YES | YES | YES | YES | YES | YES | YES | YES | YES | VALUABLE |
| *Getachew M,Abay M,Zelalem H,Gebremedhin T,Grum T,Bayray A* | YES | YES | YES | YES | YES | YES | YES | YES | NOT SURE | VALUABLE |
| *Rocha-Jiménez T,Morales-Miranda S,Fernández-Casanueva C,Brouwer KC,Goldenberg SM* | YES | YES | YES | YES | YES | YES | YES | YES | YES | VALUABLE |
| *Al-Rousan T,Schwabkey Z,Jirmanus L,Nelson BD* | YES | YES | YES | YES | YES | YES | YES | YES | YES | VALUABLE |
| *West L,Isotta-day H,Ba-break M,Morgan R* | YES | YES | YES | YES | YES | YES | NOT SURE | YES | YES | VALUABLE |
| *Tanabe M,Nagujjah Y,Rimal N,Bukania F,Krause S* | YES | YES | YES | YES | YES | YES | YES | YES | YES | VALUABLE |
| *Webber G,Spitzer D,Somrongthong R,Dat TC,Kounnavongsa S* | YES | YES | YES | YES | YES | YES | NOT SURE | YES | YES | VALUABLE |
